# Optimization and Characterization of Protein Nanoparticles for the Targeted and Smart Delivery of Cytochrome c to Non-Small Cell Lung Carcinoma

**DOI:** 10.3390/cancers12051215

**Published:** 2020-05-13

**Authors:** Vanessa Barcelo-Bovea, Irivette Dominguez-Martinez, Freisa Joaquin-Ovalle, Luis A. Amador, Elizabeth Castro-Rivera, Kristofer Medina-Álvarez, Anthony McGoron, Kai Griebenow, Yancy Ferrer-Acosta

**Affiliations:** 1 Department of Chemistry, University of Puerto Rico, San Juan 00925, Puerto Rico; vanessabarcelo1@gmail.com (V.B.-B.); irivette.dominguez@upr.edu (I.D.-M.); freisa@gmail.com (F.J.-O.); amador26@gmail.com (L.A.A.); kai.griebenow@gmail.com (K.G.); 2 Molecular Sciences Research Center, San Juan 00926, Puerto Rico; 3 Department of Neuroscience, Universidad Central del Caribe, Bayamon 00956, Puerto Rico; 413ecastro@uccaribe.edu (E.C.-R.); 118kmedina@uccaribe.edu (K.M.-Á.); 4 Department of Biomedical Engineering, Florida International University, Miami, FL 33174, USA; mcgoron@fiu.edu

**Keywords:** cancer, cytochrome c, drug delivery, Lewis Lung Carcinoma, nanoprecipitation, non-small cell lung carcinoma

## Abstract

The delivery of Cytochrome c (Cyt c) to the cytosol stimulates apoptosis in cells where its release from mitochondria and apoptotic induction is inhibited. We developed a drug delivery system consisting of Cyt c nanoparticles decorated with folate-poly(ethylene glycol)-poly(lactic-co-glycolic acid)-thiol (FA-PEG-PLGA-SH) to deliver Cyt c into cancer cells and tested their targeting in the Lewis Lung Carcinoma (LLC) mouse model. Cyt c-PLGA-PEG-FA nanoparticles (NPs) of 253 ± 55 and 354 ± 11 nm were obtained by Cyt c nanoprecipitation, followed by surface decoration with the co-polymer SH-PLGA-PEG-FA. The internalization of Cyt c-PLGA-PEG-FA nanoparticles (NPs) in LLC cells was confirmed by confocal microscopy. NP caspase activation was more efficient than the NP-free formulation. Caspase activity assays showed NPs retained 88–96% Cyt c activity. The NP formulations were more effective in decreasing LLC cell viability than NP-free formulation, with IC_50_ 49.2 to 70.1 μg/mL versus 129.5 μg/mL, respectively. Our NP system proved to be thrice as selective towards cancerous than normal cells. In vivo studies using near infrared-tagged nanoparticles show accumulation in mouse LLC tumor 5 min post-injection. In conclusion, our NP delivery system for Cyt c shows superiority over the NP-free formulation and reaches a folic acid-overexpressing tumor in an immune-competent animal model.

## 1. Introduction

Apoptosis evasion is one of the hallmarks in all cancer types [1]. The mitochondrial apoptosis pathway has been extensively studied and is known to be the most commonly hindered apoptotic pathway in cancer cells. Cancerous cells can acquire adaptations to avoid cell death, such as inhibition of mitochondrial outer membrane permeabilization [2], overexpression of survival-promoting proteins [3,4], and caspase inhibition, among others. In recent years, the induction of intrinsic or extrinsic apoptotic pathways has been a strategy in the development of new cancer treatments [5]. Cytochrome c (Cyt c) release is one key event of the mitochondrial apoptotic pathway that triggers the caspase activation cascade [6]. Deficiency of Cyt c has been shown to prevent proper apoptosome formation, inhibiting cell death and promoting cancer [7,8]. Several studies have shown that the direct external delivery of Cyt c to the cell cytosol can activate the caspase cascade and induce apoptosis in a dose-dependent manner [9,10]. Our group and others have developed several drug delivery systems for Cyt c, which can induce apoptosis selectively in different cancer types by targeting the mitochondrial apoptotic pathway [11,12,13,14,15,16,17]. These systems were designed to specifically release the drugs under reducing conditions like the ones found in the cellular cytosol, but not outside the cell, increasing their effective dose and lowering off-targets and premature drug release [18].

One feature that has been used to target cancer cells is their surface overexpression of the folic acid receptor alpha (FR). Folic acid (FA) is involved in DNA biosynthesis, and because of their rapid proliferation, changes in cell morphology, and tumor microenvironment, cancer cells have increased demands for FA compared to healthy cells, and thus overexpress the FR. Many cancer therapies have been developed using the FR as a target to enhance cancer detection. This receptor has the advantage of being internalized after interaction with its ligand, and it is recycled back to the surface, providing a constant enrichment of the drug target in the extracellular membrane of cancerous cells [19]. Once the FR-targeting drug is internalized, to release drugs selectively within the intracellular (reduced) environment, numerous delivery systems have taken advantage of the difference in glutathione (reducing agent) concentration between the internal and external cellular milieu [19].

Protein nanoprecipitation is a structure- and activity-preserving technique that permits the synthesis of Cyt c nanoparticles (NPs) with high induction of caspase activation [17]. Around 80–90% of the caspase activity is retained in Cyt c NPs compared to the native protein (100% activity) in cell-free caspase assays [12,13,16,17]. Taking advantage of these systems and their potential for nanotechnology applications in cancer treatment [20], we developed a drug delivery system for Cyt c. The system consists of Cyt c-based NPs decorated on their surface with the amphiphilic co-polymer folate-poly(ethylene glycol)-poly (lactic-co-glycolic acid)-thiol (FA-PEG-PLGA-SH). The FA moiety of the NPs targets specifically cells overexpressing the FR. After cellular uptake, the disulfide bond that covalently attaches the co-polymer to the Cyt c NPs is cleaved (in a reduced intracellular environment), Cyt c is released, and apoptosis is induced [15]. This system offers higher loading capacity in comparison to other nanocarriers because the core of the nanoparticle is made 100% of Cyt c, which is the drug itself. Our group has proved, in previous studies, the selectivity and effectivity of the decorated NPs in vitro, in HeLa cells, and in vivo, in glioma tumors [12,15]. In this study, we demonstrated the superiority of the system when compared to an individual, FA-tagged Cyt c system (NP-free system). We also showed the optimization of the NPs synthesis procedure to reduce the NPs diameter and enhance tumor entry when applied systemically. Finally, we tested the improved system in vitro in Lewis Lung Carcinoma (LLC) cells, and in vivo using a syngeneic (immune-competent) mouse model, which is an effective approach for studying how NP therapies reach their target in the presence of a functional immune system [21].

## 2. Results

### 2.1. Folic Acid Uptake by LLC Cells

**Figure 1:**
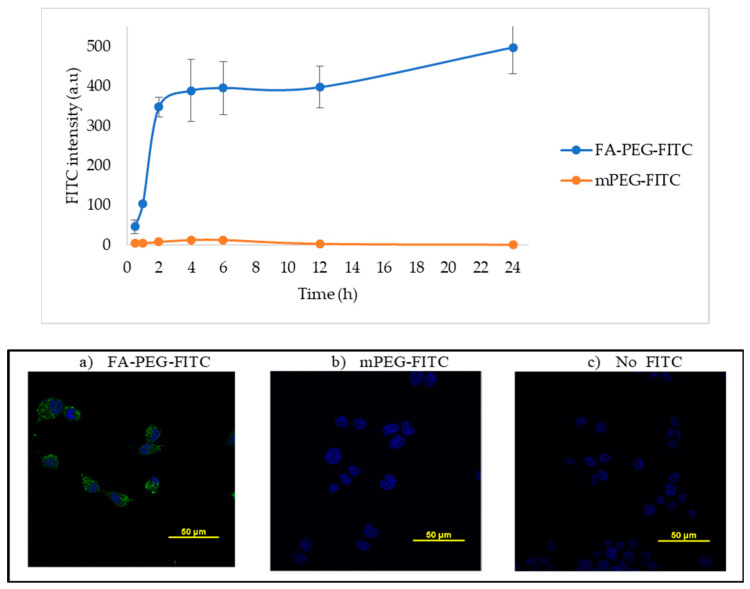
Lewis Lung Carcinoma (LLC) cell uptake of folic acid modified with FITC. Upper panel—the intensity of FITC fluorescence signal (Ex. 488 nm) in fixed LLC cells incubated with 50 µM of FA-PEG-FITC and FITC-PEGm; m = methoxy control, measured by confocal microscopy at several time points and plotted. Lower panel—representative images of LLC cells after 6 h of incubation with (**a**) FA-PEG-FITC, (**b**) mPEG-FITC, or (**c**) no FITC control. Nuclear stain DAPI shown in blue and FITC in green. FITC intensity was calculated by measuring the pixel intensity of each image using Image J, n = 9.

**Figure 2:**
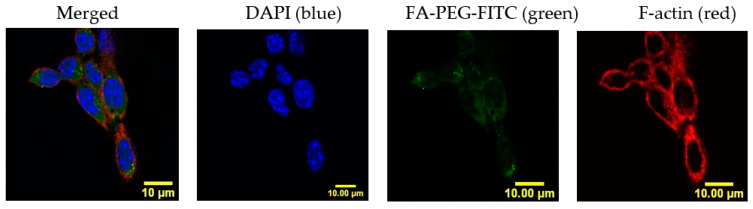
Folic acid distributes in the LLC cell cytosol after uptake. Confocal imaging of LLC cells labeled with cytoskeletal F-actin (red) and nucleus (DAPI, blue) confirm subcellular localization of FA-PEG-FITC (green) throughout the cytosol after 1 h of incubation. Scale bar: 10 μm.

Cellular uptake of NPs can be compromised by size, hydrophobicity, and lack of target of the NP, among other factors [22]. In our system, we addressed this challenge by adding a FA ligand in the surface of the NP to induce FA receptor-mediated endocytosis. To confirm if LLC cells could carry out this process, we studied the FA internalization kinetics by confocal microscopy. LLC cells were incubated in RPMI medium with no folic acid for a period of 24 h, with either 50 µM of a fluorescently labeled FA construct (FA-PEG-FITC) or a binding specificity control without FA (methoxy-PEG-FITC). Cells were washed and fixed at several time-points (0.5, 1, 2, 4, 6, 12, and 24 h). The fluorescence signal intensity of FITC (488 nm) was quantified per cell and compared between the two constructs. Figure 1 shows that FA internalization steadily increased for a period of 2 h, where it reached a plateau. After this time point, the levels of FA detected remained constant, probably due to saturation/recycling of the FR. Incubation with the methoxy-PEG-FITC (mPEG-FITC) control showed no intracellular fluorescence signal. These results support that LLC cells can selectively uptake FA because the fluorescence signal was detected within the cells exposed to FA-PEG-FITC, having the ligand that binds the FR for subsequent internalization, but not the mPEG-FITC (Figure 1). Figure 2 shows an image of FA-PEG-FITC uptake by LLC cells after a 1 h incubation. The LLC cell cytoskeleton, stained with F-Actin (in red), illustrates the internalization of FA-PEG-FITC (green) and its distribution within the cytosol.

### 2.2. Synthesis and Characterization of Nanoparticle-Free Cyt c Formulation: Cyt c-PEG-FA

To test the advantage of using a targeted nanocarrier over individual molecules of Cyt c, we constructed a Cyt c-PEG-FA bioconjugate (no NPs). PEG-FA was conjugated to Cyt c lysine groups using the crosslinker succinimidyl-3-(2-pyridyldithio) propionate (SPDP). SPDP is used to conjugate amine-to-sulfhydryls via NHS-ester and pyridyldithiol reactive groups that form cleavable, reducible disulfide bonds with cysteine sulfhydryls. To ensure that there was at least 1 mol of crosslinker per mol of Cyt c, a 1:8 Cyt c:SPDP molar ratio was used in the reaction, which resulted in a final modification of 2 moles of SPDP per mol of Cyt c. Next, the activated Cyt c-SPDP intermediate was reacted with 8 equivalents of PEG-FA, resulting in a final modification of 2 moles PEG-FA per each mol of Cyt c (Table 1).

**Figure 3:**
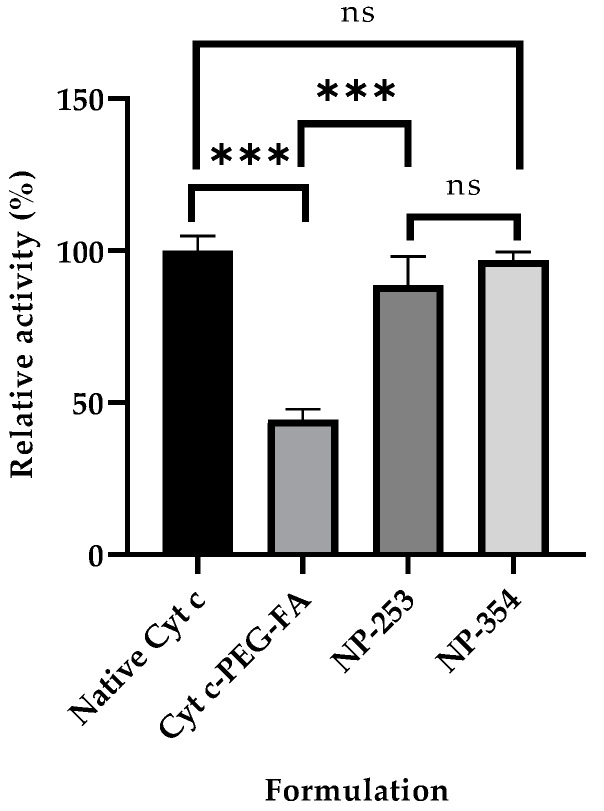
Caspase activity of surface-decorated nanoparticles (NPs) and NP-free formulation using a cell-free assay. The relative caspase activities for NP-253 and NP-354 surface-decorated NPs were not significantly different between them or when compared to the native Cyt c (ns compared to both NPs). The NP-free formulation, Cyt c-PEG-FA, showed decreased caspase activity compared to both NP-253 and NP-354 (*** *p* < 0.001, *n* = 3 for each), and native Cyt c (*** *p* < 0.001, *n* = 3). Error bars represent the calculated standard deviations (SD). The names given to the nanoparticles, NP-253 and NP-354, refer to Cyt c-PLGA-PEG-FA NPs with diameters of 253 and 354 nm, respectively.

Cell-free caspase 3, 7, and 10 activity assays were made to determine the effect of Cyt c modification with PEG-FA on its activity. Our results showed that the final product of individual molecules of Cyt c tagged with FA retained 44 ± 3.4% of the activity in comparison to native Cyt c (Figure 3). This activity loss has already been reported for Cyt c, and it mainly is due to a change in the tertiary structure of Cyt c after crosslinking to amine groups, which can lead to a reduction of its activity [23].

### 2.3. Cyt c Nanoprecipitation Optimization and Characterization (Synthesis of Cyt c Nanoparticles)

A reduction in the NP size has been shown to help the diffusional hindrance and improve the chances for in vivo tumor penetration [24]. Aiming to reduce the diameter of the previously reported Cyt c-PLGA-PEG-FA NPs (338 ± 8 nm) [12], four parameters of the nanoprecipitation step were examined: Cyt c solvent, anti-solvent, solvent:anti-solvent ratio, and Cyt c concentration. Four solvents were tested to determine which one was the best to dissolve Cyt c completely and further induce its nanoprecipitation: deionized (DI) water, NaCl 10 mM, methyl-ß-cyclodextrine solution, and 10 mM PBS buffer pH 7.4. The best among the tested solvents to induce Cyt c nanoprecipitation was DI water. Dissolving Cyt c in NaCl 10 mM, methyl-ß-cyclodextrine solution, and 10 mM PBS buffer pH 7.4 induced the formation of aggregates. Also, Dynamic Light Scattering (DLS) measurements were not reliable for the Cyt c NPs obtained using these three last solvents due to aggregate formation. Subsequent nanoprecipitation of Cyt c for NP optimization was performed using DI water as the solvent.

During the nanoprecipitation step, the dissolved Cyt c in DI water was mixed with an anti-solvent to obtain an “emulsion” composed of our Cyt c NPs. Thus, we next tested four different anti-solvents (THF, acetonitrile, acetone, and ethanol) to determine if they could differentially affect NP diameter. Acetonitrile had been previously used to generate the Cyt c NPs by our group [12]. The use of tetrahydrofuran (THF) resulted in an increase of the Cyt c NPs diameter compared to the NPs formed using acetonitrile (from 163 ± 13 to 219 ± 9 nm) at a Cyt c concentration of 5 mg/mL. The use of acetone and ethanol did not precipitate Cyt c despite Cyt c being insoluble in these two anti-solvents (Table 2). Therefore, our results showed that the best anti-solvent to generate Cyt c NPs was (still) acetonitrile.

Using the best solvent and anti-solvent combination according to the above results (DI water and acetonitrile) we tested the effect of Cyt c starting concentration during nanoprecipitation. NP diameter and polydispersity index (PDI) were reduced by lowering Cyt c concentration from 10 to 5 mg/mL (35% reduction in NP diameter and 51% reduction in PDI) (Table 3). There was no statistical difference between the NP diameters obtained using 2.5 and 5 mg/mL Cyt c solutions. When comparing the Cyt c precipitation efficiency or residual caspase activity of NPs obtained from 5 and 10 mg/mL, no statistically significant difference was found using ordinary one-way ANOVA and multiple comparison Tukey’s test (95% confidence interval). Caspase 3, 7, and 10 activation by Cyt c NPs remained 88 ± 2% for NP-253 and 96 ± 3% for NP-354, with no significant difference when compared to the native protein, showing an almost intact activity (Figure 3 and Table 3).

**Figure 4:**
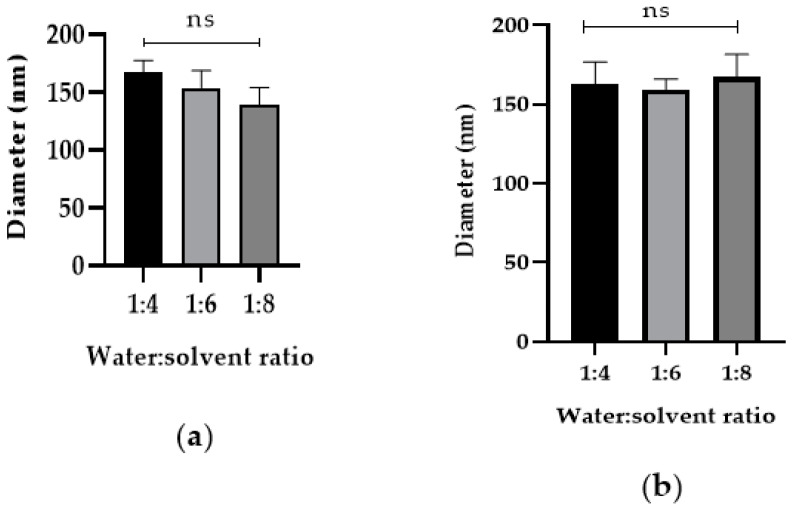
Increased addition of co-solvent during nanoprecipitation does not affect Cyt c NP diameter. Cyt c NPs were obtained by adding three different ratios of DI water-to-acetonitrile (1:4, 1:6, and 1:8) with two different starting Cyt c concentrations: (**a**) 2.5 mg/mL, left panel and (**b**) 5 mg/mL, right panel. One-way ANOVA tests were not significant among all conditions. Data are the averages of three preparations, and the error bars show the SD.

After determining that DI water–acetonitrile was the best solvent and anti-solvent combination, we tested if changing the DI water-to-acetonitrile ratio affected NP diameter. The addition of higher acetonitrile in the DI water to acetonitrile ratios 1:4, 1:6, and 1:8 during nanoprecipitation did not affect the Cyt c NP diameter, as shown in Figure 4. Our results show that, from the four approaches tested, reduction of protein concentration was the only one that helped decrease Cyt c NPs diameter (Figure 3).

### 2.4. Cyt c-PLGA-PEG-FA NP Optimization and Characterization

**Figure 5:**
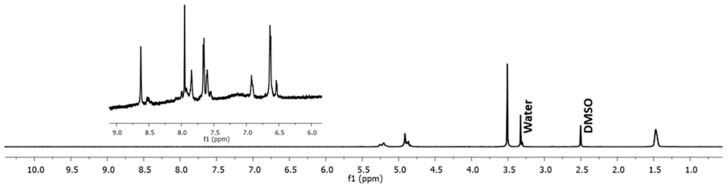
H^1^ NMR spectrum of the copolymer SH-PLGA-PEG-FA. The peaks between 9 and 6 ppm correspond to FA protons, while the peaks between 6 and 1 ppm correspond to both PLGA and PEG.

In the previous experiments, several methods to induce a reduction of the Cyt c NPs core size were tested as part of the optimization procedures. Following Cyt c NPs preparation, the synthesis of the polymer used to decorate the NPs, SH-PLGA-PEG-FA, was made employing the reaction conditions reported by Morales-Cruz et al. (2014) [17]. To confirm the reproducibility of the polymer synthesis, ^1^H NMR of the SH-PLGA-PEG-FA was performed. The ^1^H NMR spectrum of the copolymer SH-PLGA-PEG-FA confirmed the conjugation of SH-PLGA and COOH-PEG-FA (Figure 5). The peaks between 9 and 6 ppm corresponded to the aromatic protons in FA, while the peaks between 6 and 1 ppm corresponded to both PLGA and PEG, validating the reproducibility of polymer synthesis.

After nanoprecipitation, Cyt c NPs with three different diameters (139, 163, and 251 nm, generated from Cyt c solutions of 2.5, 5, and 10 mg/mL, respectively) were obtained. Cyt c NPs alone (without decoration) were found to be stable only in the mix of DI water:acetonitrile in which they were formed (at least in a 1:4 ratio), but not in aqueous solution alone. Cyt c NPs were subjected to surface modification with the crosslinker SPDP, followed by the conjugation of the co-polymer SH-PLGA-PEG-FA. After modification, Cyt c NPs with diameters of 163 and 251 nm remained stable after being transferred to the aqueous solution, while the 139 nm NPs dissolved (Table 4). The hydrophobic PLGA segment of SH-PLGA-PEG-FA protects Cyt c NPs from dissociating in aqueous solution, while the hydrophilic PEG segment allows Cyt c NPs to solubilize in aqueous solution. The smaller the particles, the less surface protection is afforded by the polymer due to increased curvature; thus, it might be necessary to find other strategies to afford smaller NPs for such applications.

Characterization of the Cyt c NPs and Cyt c-PLGA-PEG-FA NPs was made using Dynamic Light Scattering (DLS), zeta-potential, UV-spectroscopy, and Scanning Electron Microscopy (SEM). Table 4 shows the properties of the Cyt c-PLGA-PEG-FA NPs obtained from undecorated Cyt c NPs with diameters of 163 and 251 nm that were further modified. After PLGA-PEG-FA conjugation, the initial diameter of the Cyt c NPs generated with different Cyt c concentrations increased around 100 nm in both cases. Multiple comparison analysis showed a statistical difference between the diameters of NP-253 and NP-354 (*p* < 0.001). There were no significant differences in encapsulation efficiency, actual loading capacity, and zeta potential between Cyt c-PLGA-PEG-FA NP obtained using 5 mg/mL (NP-253) and 10 mg/mL (NP-354) Cyt c during nanoprecipitation.

**Figure 6:**
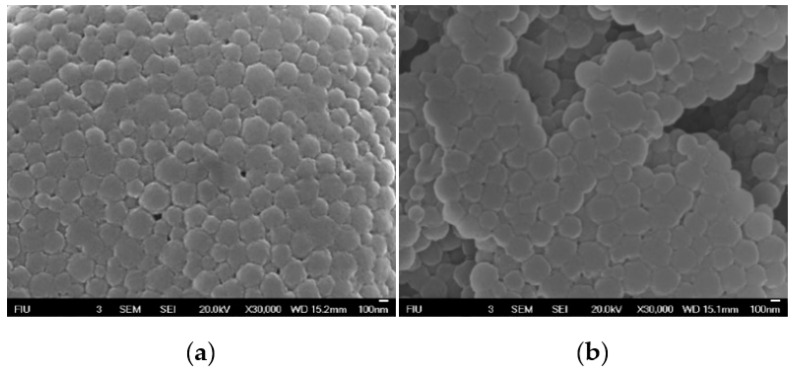
Scanning electron micrographs of Cyt c-PLGA-PEG NPs. (**a**) NP-253 and (**b**) NP-354. Scale bar in a and b:100 nm.

To confirm the morphology of the decorated NPs, these were visualized using Scanning Electron microscopy (SEM) (Figure 6). Results showed spherical particles with a consistent size distribution for both NP-253 and NP-354, reproducing the ones previously produced by Morales-Cruz et al. (2004) [17], but with different sizes.

### 2.5. Release Profile of Nanoparticles

**Figure 7:**
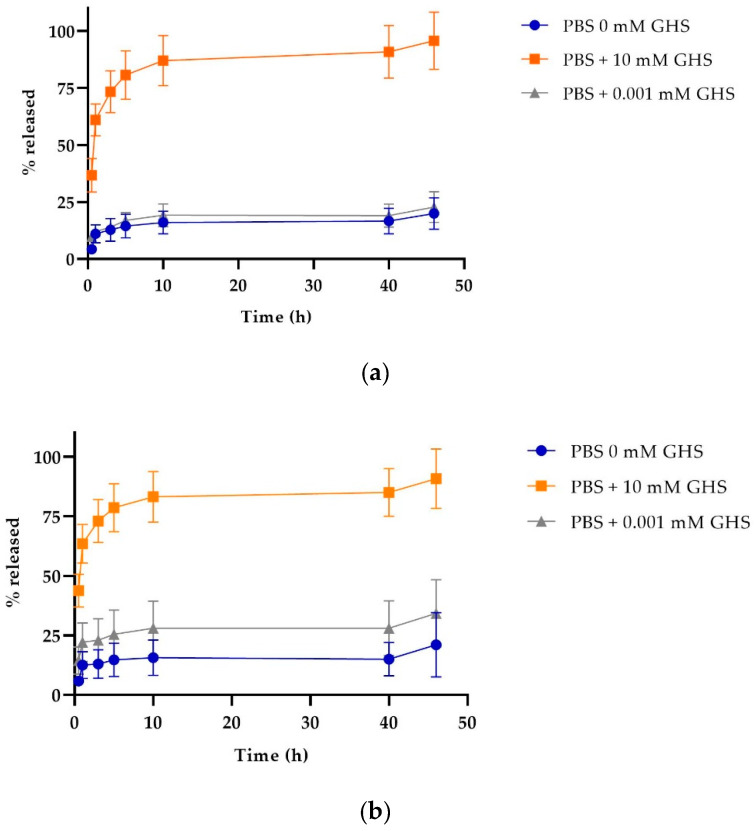
Cytochrome c release profile of nanoparticles under reducing (intracellular) and non-reducing (extracellular) conditions. Cyt c release profile for (**a**) NP-253, top and (**b**) NP-354, bottom. The data are the averages of three release experiments, and the error bars are the calculated SD.

One of the characteristics that make these NPs a smart targeted drug delivery system is their capacity to respond to stimuli, such as changes in pH. The SPDP crosslinker enables NPs to detach their stabilizing PLGA-PEG-FA polymer decoration once they are in contact with a reducing environment, destabilizing the NP and releasing their Cyt c content. Figure 7 shows the release profiles of Cyt c-PLGA-PEG-FA NPs (both NP-354 and NP-253) under different reducing conditions, emulating the cell’s intracellular (glutathione reducing agent added) or extracellular environment (no glutathione). In both NPs, the optimum release condition was PBS pH 7.4 containing 10 mM glutathione S-transferase (GHS), which simulates the reducing environment inside the cells. Times for 50% NP Cyt c release were 1 and 2 h for more than a 75% discharge. The release behaviors of NP-253 and NP-354 under reducing conditions were not significantly different between them according to an ordinary one-way ANOVA multiple comparisons analysis (*p* > 0.9). Both NP-253 and NP-354 had significantly lower release under the conditions that simulate the extracellular physiological environment (PBS + 0.001 mM GHS or PBS alone) [25] in comparison to PBS + 10 mM GHS (*p* < 0.0001). The difference between 0 and 0.001 mM GHS was not significant for both of the NP formulations. These results show that, due to their stimuli-responsive controlled release, the Cyt c-PLGA-PEG-FA NPs can keep the cargo (Cyt c) encapsulated while they are in an environment like the bloodstream and can deliver it when the system is internalized by cells.

### 2.6. Cell Viability and Target Specificity Studies

**Figure 8:**
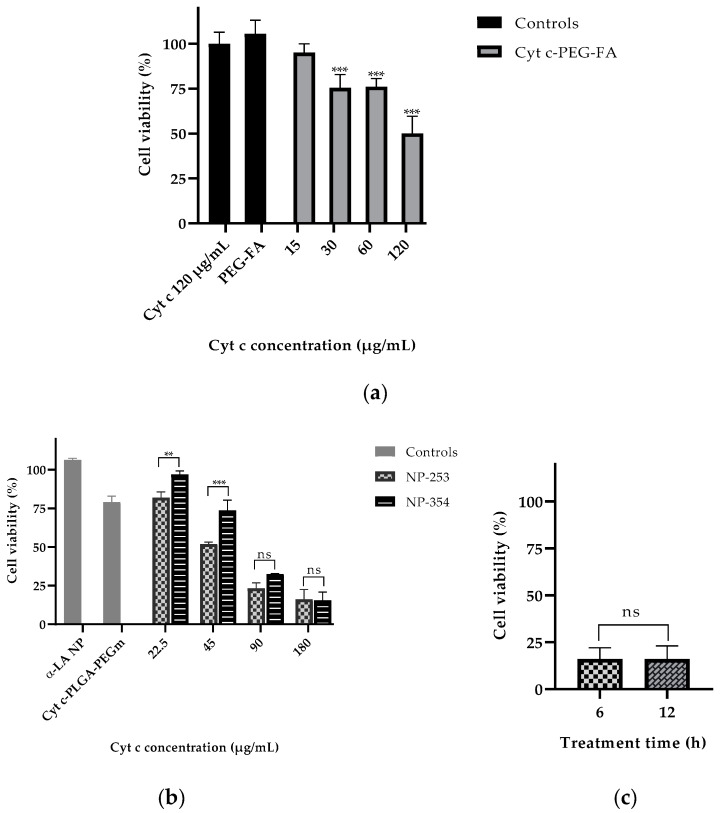
Cytochrome c delivery in targeted NPs efficiently reduces LLC cell viability after 6 h in a concentration-dependent manner. (**a**) LLC cell viability assay (MTS) after 6 h of treatment with Cyt c alone (120 μg/mL) and PEG-FA (120 μg/mL) as controls compared to the NP-free formulation Cyt c-PEG-FA at different concentrations (*** *p* < 0.001, *n* = 6). (**b**) LLC viability assay after 6 h of treatment with NPs made with non-apoptotic protein α-Lactalbumin (αLA NP) and Cyt c-PLGA-PEG-m (m = methoxy) as controls, compared to the NP formulations NP-253 and NP-354 (** *p* = 0.009 and *** *p* < 0.001, *n* = 3). (**c**) LLC cell viability assay after 6 h and 12 h of treatment with NP-253 (180 µg/mL). Error bars represent the calculated SD.

To test the effectiveness of the NP-free versus NP-containing system in lung carcinoma cell viability, LLC cells were incubated with either the NP-free formulation (Cyt c-PEG-FA) (Figure 8a) or NP formulation (Cyt c-PLGA-PEG-FA, both NP-253 and NP-354) (Figure 8b). The NP-free formulation, Cyt c-PEG-FA, reduced LLC cell viability up to 50% in a dose-dependent manner after 6 h of incubation, with an IC_50_ of 129.5 μg/mL (R^2^ = 0.8386), in comparison to non-treated cells. Neither Cyt c alone nor the polymer PEG-FA had any effect on cell viability, supporting that the covalent attachment of PEG-FA to Cyt c allowed Cyt c to induce apoptosis in LLC cells (Figure 8a).

The NP formulations, NP-253 and NP-354, both decreased LLC cell viability in a dose-dependent manner (Figure 8b). The NP-253 had an IC_50_ at a concentration of 49.2 µg/mL (R^2^ = 0.9781), which showed to be more efficient at decreasing cell viability than NP-354 with an IC_50_ = 70.1 µg/mL (R^2^ = 0.9781). Therefore, NP-253 and NP-354 formulations showed to be 2.6 and 1.8 times more cytotoxic than the NP-free formulation, which is also a system that can significantly decrease cell viability, but less effectively. Because of its improved performance after 6 h of treatment in LLC cells, we tested the effect of NP-253 after 12 h. However, there was no further improvement in the outcome (Figure 8c). These results confirm that the NP-formulation can successfully deliver inside the cells functional Cyt c proteins with an intact structure and activity.

**Figure 9:**
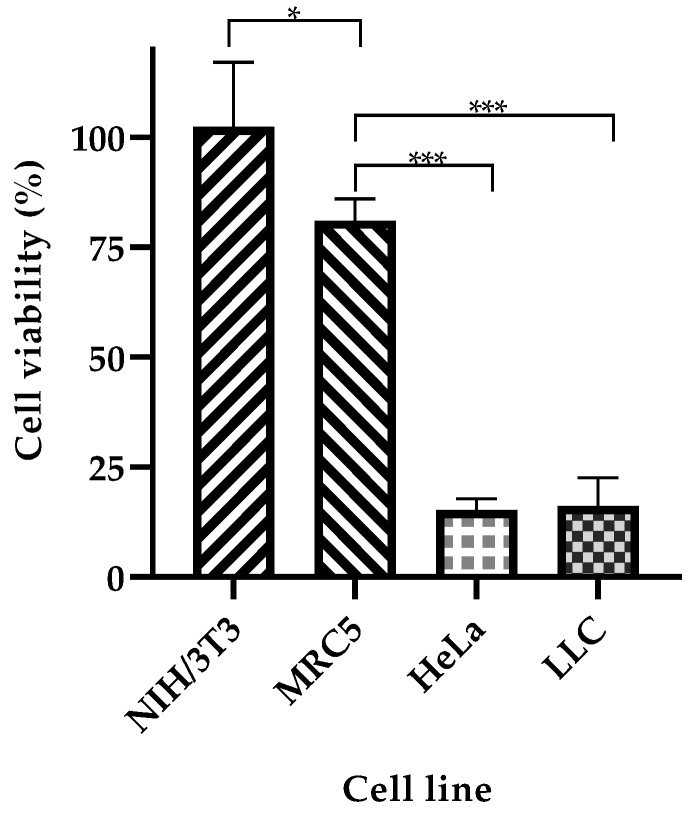
NP-253 decreases the viability of cancerous cell lines compared to non-cancerous cells. Cell viability after treating cancerous (LLC and HeLa cells) and non-cancerous (NIH/3T3 and MRC-5) cells with NP-253 (180 µg/mL of Cyt c) for 6h. One-way ANOVA, * *p* = 0.01 and *** *p* < 0.001, respectively, *n* = 3. Error bars represent the calculated SD.

Next, we tested the selectivity of our system towards cancer cells. NP-253 reduced the viability of LLC cells and HeLa cells (cervical cancer), both overexpressing FR (Figure 9). NIH/3T3 (mouse embryonic fibroblasts) and MRC-5 (human fibroblasts derived from lung tissue) cell lines were used as healthy control cells that did not overexpress FR. The viability of NIH/3T3 cells was not affected after incubation with NP-253, and MRC-5 cell viability slightly decreased after incubation with the same NP. Nevertheless, the decrease in cell viability of cancer cells after NP-253 was, on average, fourfold. These results show that the targeted NP-253 drug delivery system is selective towards FR-expressing cancer cells.

### 2.7. Study of Cell Death Induction Using DAPI and Propidium Iodide Co-Localization

**Figure 10:**
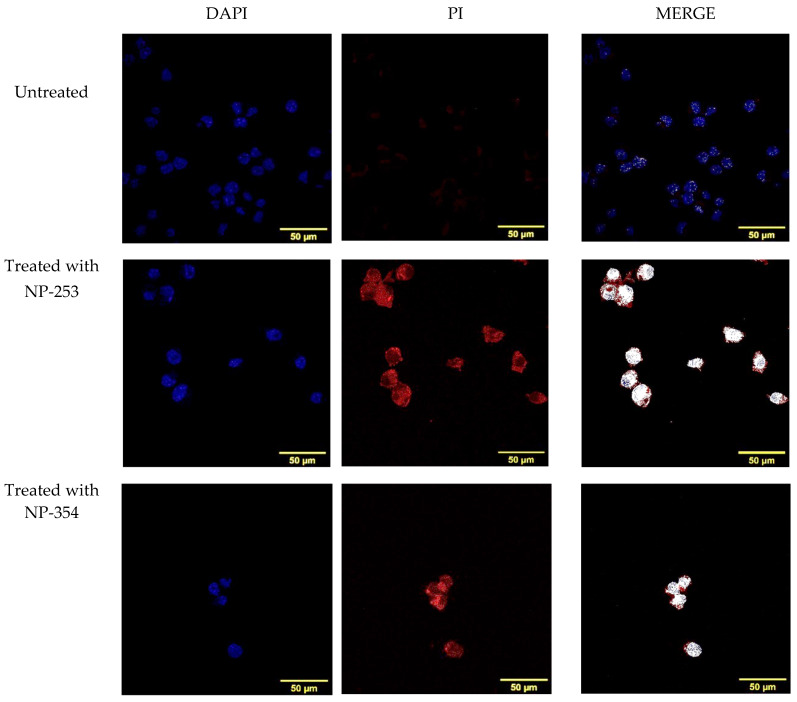
Induction of cell death in LLC cells by NP-253 and NP-354 confirmed by nuclear PI staining. Immunofluorescence of untreated LLC cells (upper panel) shows no co-localization of nuclear DNA dye DAPI (blue) with the cell death (late apoptosis) marker propidium iodide (PI, red), as shown in the upper right panel (merge, white). LLC cells treated 6 h with either NP-253 (middle panel) or NP-354 (lower panel) show co-localization of DAPI and PI (right panels, white color shows co-localization), suggesting compromised membranes and entry of PI inside the nucleus.

To confirm if the Cyt c released from NP-253 and NP-354 was structurally functional and induced cell death in LLC cells, we used a second additional method to MTS, propidium iodide (PI). PI is a healthy-cell impermeable and DNA-binding fluorescent stain, which enters the cell when its membrane is compromised. Figure 10 shows the co-localization of PI in the nucleus of cells treated with both NP-253 and NP-354 formulations, but not in untreated cells. Because PI fluorescence can only be found inside the cells with compromised membranes that are going through a cell-death process, this marker is used to label cells during late apoptosis. These results suggest that cells exposed to the NPs could be decreasing their viability through induction of the apoptotic pathway by Cyt c. This result is also supported by the data showing that the NP-decorated polymers were not toxic to the cells, leaving Cyt c as the only potential cell death inducer.

### 2.8. Cyt c-PEG-FA and NP-253 Internalization

**Figure 11:**
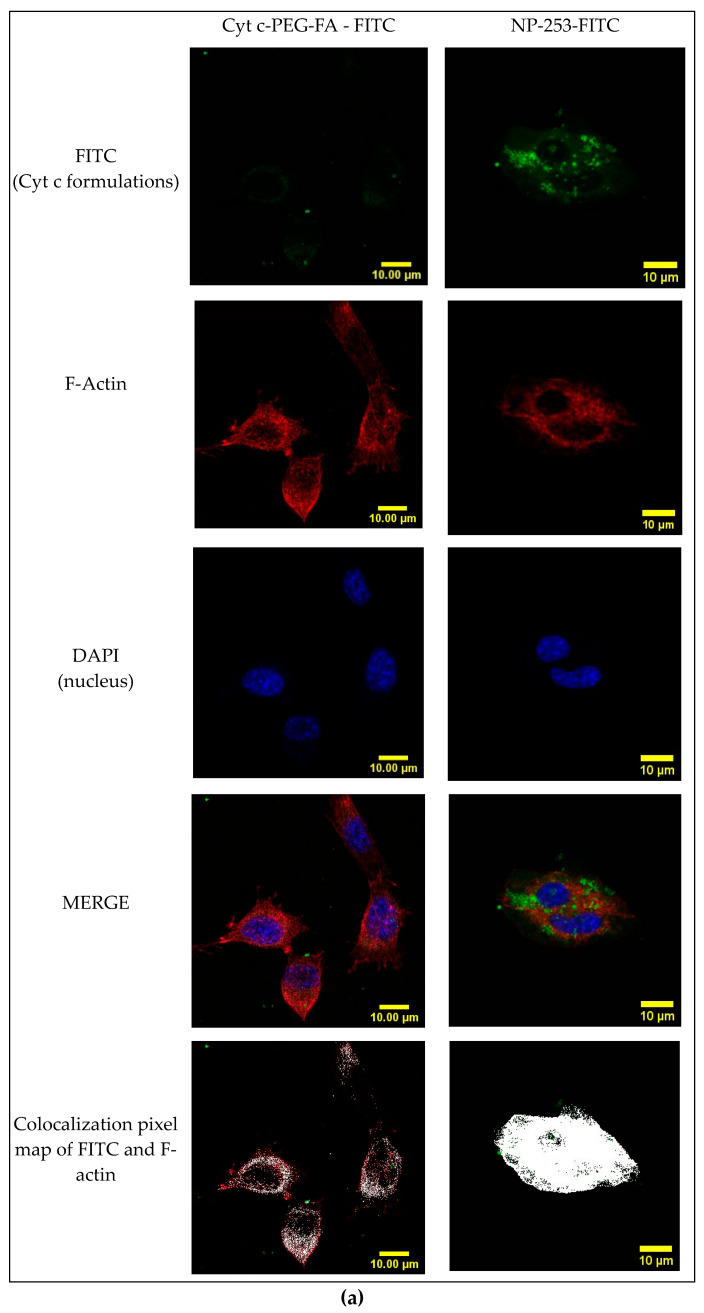

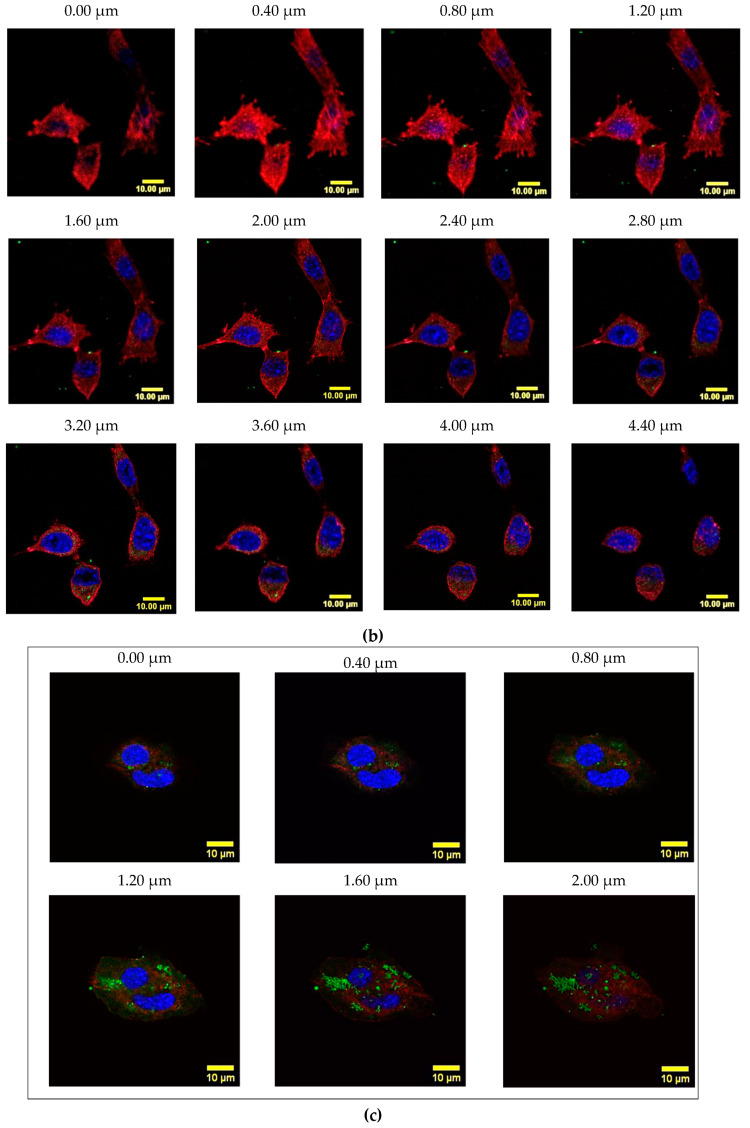
Internalization of FITC-labeled NP-253 and Cyt c-PEG-FA by LLC cells. (**a**) Confocal images of LLC cells treated with FITC-labeled Cyt c-PEG-FA and NP-253 (green) after 6 h incubation. The images shown in the lower panel are the co-localized pixels of FITC (green) and F-actin (red), which are shown in white. Nuclear stain DAPI is shown in blue. (**b**) Consecutive Z-stack images (cell bottom to top) of FITC-labeled Cyt c-PEG-FA (100 µg/mL) treated LLC cells. (**c**) Consecutive Z-stack images (cell bottom to top) of FITC-labeled NP-253 (35 µg/mL) treated LLC cells.

To determine if the FA-containing formulations could be internalized by FR-overexpressing cells, LLCs were incubated with FITC-conjugated Cyt c-PEG-FA and NP-253 for 6 h (Figure 11a). The presence of FITC signal within the cytosol is shown by confocal Z-stack images in the Cyt c-PEG-FA-FITC (NP-free control, Figure 11b) and the NP-253-FITC (Figure 11c), indicating that both formulations were internalized by LLC cells. Nevertheless, the NP formulation showed increased uptake of FA compared to NP-free formulation, possibly because it holds more FA-moieties per unit than the NP-free formulation, increasing its probabilities to be endocytosed. These results are comparable to studies performed in different FR-overexpressing cell lines, such as HeLa [23]. Studies show that the binding and uptake of FA attached to other molecules like FITC does not affect the interaction of FA with the FR [23]. Therefore, our NP internalization results are a valid representation and reproduce the kinetics of the folic acid receptor FA-internalization in FR-overexpressing LLC cells.

### 2.9. In Vivo Imaging Studies of NP Biodistribution in a Lung Carcinoma Mouse Model

**Figure 12:**
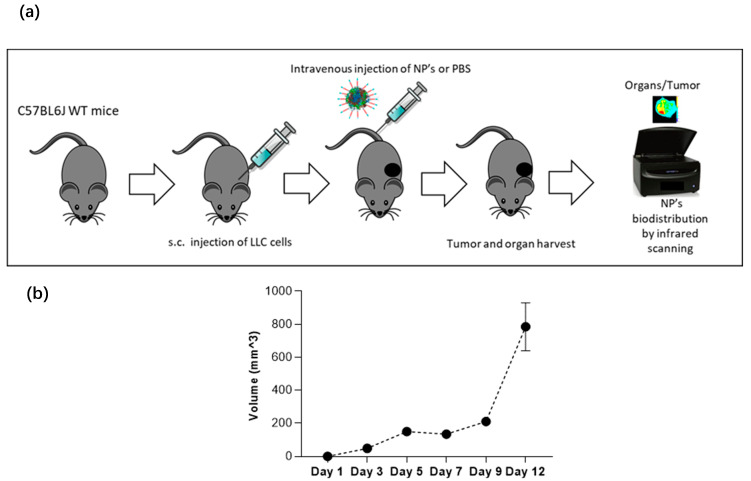
Syngeneic Lewis Lung Carcinoma mouse model. (**a**) C57BL6J wild-type mice were injected subcutaneously with the LLC cell line for an average of 10 d of tumor growth, and 0.5 mg of NPs were injected via tail vein to 25–28 g male mice. After NP injection, mouse tumor and organs were harvested for infrared scanning. (**b**) In this carcinoma mouse model, tumor volume increases in time, and progression is shown from day 1 to day 12.

**Figure 13:**
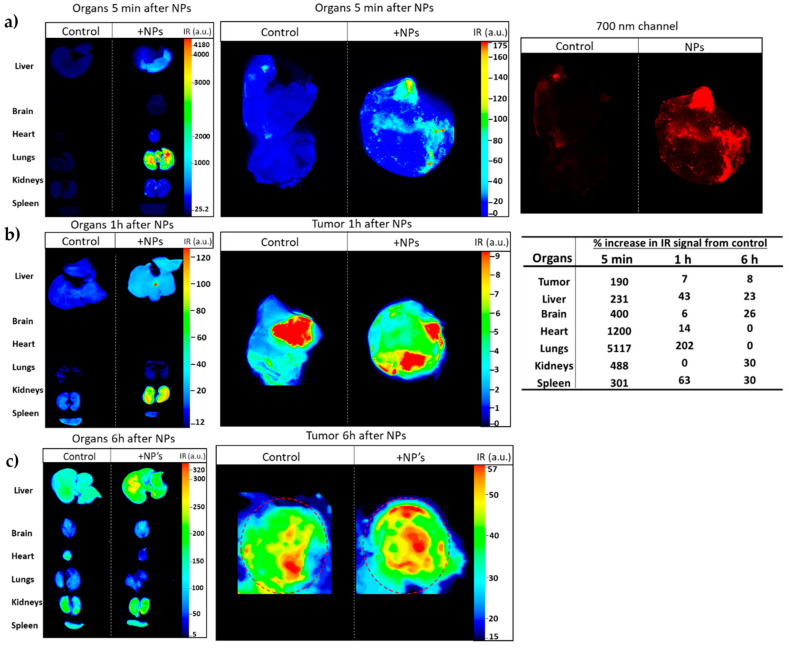
The IR-labeled NP-253 reaches the tumor area in a Lewis Lung Carcinoma mouse model. (**a**) Upper panel left and middle images show in multicolor the 700 nm signal intensity (heat map of IR signal intensity areas), representing NP-253 distribution in mouse tumor and organs at 5 min after intravenous (i.v.) injection of NP or control PBS (also having IR background signal). The far-right panel shows the infrared signal from the NPs in the tumor 5 min after i.v. injection only with the red channel (for clarity of NPs distribution in tumor). (**b**,**c**) Middle and lower panels show a heat map of the IR signal from the NPs or PBS control in the tumor 1 and 6 h (respectively). The table on the right shows the percent of IR signal (IR pixels) from the tissues of NP-injected mice over the control mice. Each image represents one mouse, n = 6.

To assess if the NPs target lung tumors in vivo, NP-253 was crosslinked by an amine-directed crosslink to a near-infrared reactive dye (IRDye^®^ 680RD). This IR label allowed us to track the organ and tumor targeting of NPs in mice after systemic intravenous injection. The Lewis Lung Carcinoma syngeneic mouse model was used as our in vivo system of non-small cell lung carcinoma. To develop this tumor model, C56BL6J mice were injected subcutaneously (s.c.) with LLC cells on the upper back (Figure 12a,b). After an average of 10 d of tumor growth, an intravenous injection (tail vein) of near-infrared (IR)-labeled NP-253 was administered. Mice were euthanized at different time points, and organs were harvested to detect if the NPs reached the tumor area/organs, and at which time point (Figure 13). After scanning the mice on an infrared detector, a comparison in signal intensity between the control and NP-injected mice (expressed in % observed IR signal of NP-injected mice over control) showed the signal in tumor decreased as time in circulation increased: 5 min, 190%; 1 h, 7%; and 6 h, 8% IR signal. This NP distribution is indicative of compound processing and clearing throughout time (Figure 13a–c). Like typical drugs entering through the vessel network, IR NP-293 distributed systemically through the heart, lungs, and the targeted tumor site. Intravenous administration is one of the best ways for the NPs to be delivered because this route avoids the first-pass effect through the gastrointestinal tract and liver, where many drugs can bind and lose their effective dose before reaching general circulation and their target [26].

The IR-labeled NP-253 solubility was not as high as the unlabeled NPs, and their solubility had to be improved by adding the excipient trehalose. The upper right panel in Figure 13a, showing the red 700 nm channel only, demonstrates how the IR-NP-293 were not degraded or accumulated in a specific organ, but they circulated, reached the tumor site, and continued their path through different organs in the measured time. The infrared signal was obtained by using an infrared pixel quantification scanner and software (LI-COR Odyssey CLx, Image Studio ^TM^ software). This signal was observed in different mouse tissues at three different time points. At 5 min (Figure 13a), we could trace how the intravenous (i.v.)-delivered IR-NP-293 traveled from the vessel network to the pulmonary circulation (5 min: 5117%; 1 h: 202%; 6 h: 0%, IR signal over control in lung tissue). After 1 h (Figure 13b), lungs, heart, liver, and spleen retained the highest amount of IR signal, which could indicate circulating NPs that did not bind to FR started to metabolize at this time point. At 6 h after NP injection (Figure 13c), kidney, spleen, liver, and brain still showed low IR intensity, and this could be a point where the NPs were at the end of their metabolic processing. Altogether, these results show that a near-IR system crosslinked to a targeted NP system can allow detection of NP organ and tumor distribution throughout time with high sensitivity. This system also allows us to determine for how long these NPs are retained in the different tissues, how they circulate, and how long they take to dissipate from organs or tumors.

## 3. Discussion

In this work, a new method for stabilizing Cytochrome c nanoparticles by covalently coating them with the hydrophobic polymer poly(lactic-co-glycolic) acid (PLGA) was tested. PLGA is a biocompatible, biodegradable, and non-toxic polymer that has been intensely studied in the field of Drug Delivery Systems and has received FDA approval for various applications, including drug delivery [27,28,29]. The idea tested in our studies was to coat the Cyt c NPs with PLGA using a hetero-bifunctional linker, such as succinimidyl 3-(2-pyridyldithio) propionate (SPDP), which includes a disulfide bond, to be able to shed the polymer shell inside the reducing environment of the cell. The presented experiments demonstrated that the tested strategy of folate receptor targeting using a core-shell NP loaded with pro-apoptotic protein Cyt c is an efficient method to deliver this drug and induce cell death in lung carcinoma cells. The designed nanoparticles were delivered inside the LLC cells in cell culture and reached the targeted tumor site after 5 min in a lung carcinoma mouse model with an intact immune system. The biodistribution of NP-253 after an intravenous application was detected by infrared-labeling in a time-dependent manner at 1 and 6 h post intravenous administration. The optimized NPs were able to reduce lung carcinoma cell viability in the LLC murine model and in the human FR overexpressing cervical cancer HeLa cells, on average, four times more than in the non-cancerous cell lines MRC-5 or NIH/3T3 cells, demonstrating cancer-cell target specificity.

Once introduced to a physiological environment, NPs are exposed to adsorption of biomolecules, which result in the development of a layer known as the protein corona, which changes the original properties of the nanoparticle. In our system, the formation of this protein corona on the NP surface was regulated by modifying the nanoparticle’s surface with the amphiphilic co-polymer folate-poly(ethylene glycol)-poly (lactic-co-glycolic acid)-thiol (FA-PEG-PLGA-SH). Modifications with such polymers have been shown to enhance the colloidal stability and extend the circulation time in blood by enabling the NP to escape from immune system clearance [30,31]. This was the case for our NPs, which lasted in circulation in the tumor for at least 6 h after i.v. injection, according to infrared detection.

What about the metabolism of these NPs? The expected metabolites from the nanoparticles include compounds such as PEG, PLGA, Cyt c, and FA. After cellular internalization, PEG is endocytosed, and from the early endosome, PEG molecules can be recycled back to the plasma membrane to return to the circulation and finally undergo excretion by the kidney through the urine [30]. PLGA is a biodegradable polymer that is hydrolyzed into lactic acid and glycolic acid, which are used within the cell [32]. Cyt c undergoes protein catabolism inside cells and is reduced to amino acids, and FA is a regular vitamin that cells uptake and metabolize to produce purines [33]. Thus, we expect little or no toxic secondary effects after repeated intravenous injection of the NPs in mice.

Several studies using folate receptor targeting have employed NIH/3T3 and MRC-5 as control, non-cancerous cell lines that express low folic acid receptor levels [34,35]. In these studies, we demonstrated the target preference of these NPs to folic acid receptor-overexpressing cancer cell lines LLC and HeLa, over MRC-5 and NIH 3T3 cells. Our experiments using HeLa and MRC-5 also show the translational application of our NP formulation to human FR-overexpressing cancers. Uptake of NPs in cancerous murine and human cells, together with the in vivo results of tumor-targeting, show that our NPs have cancer cell specificity and tumor-targeting capability. Studies of these NPs and their effect on tumor growth are currently ongoing. We expect the NP-253 to be more effective than NP-354, as its smaller size may allow maximal biological function. Some studies have shown, when comparing different-sized NPs (50, 100, and 200 nm NPs), that the smaller NPs have increased tumor penetration and uptake by tumor cells, and the slowest tumor clearance, leading to a more extended retention period in tumor tissue over time and a better efficacy within the tumor microenvironment [36]. Nevertheless, one of the limitations of NP drug delivery systems is conveying the effective dose to induce cell death in vivo, and in that aspect, a larger nanoparticle such as the NP-354 or delivery of a more potent drug could have an advantage. Other aspects to take into consideration besides size and drug load are NPs surface charge and optimal NP delivery method (tumor-direct or systemic tumor application), which depends on the type and progress of the targeted tumor [24,37].

After chemical modification of Cyt c to the Cyt c-PEG-FA (NP-free formulation), there was a reduction in the relative caspase activity to 44%, using cell-free caspase 3, 7, and 10 activity assays, compared to the native Cyt c. The reduction of Cyt c activity after crosslinking to the amine groups has been previously reported by our group and is associated with changes in the protein’s tertiary structure [23]. The lower activity of Cyt c-PEG-FA can also be due to possible random modifications to the amine groups corresponding to Lys 7, 25, 39, and 72, which play an important role in the interaction of Cyt c with the apoptotic protease activating factor 1 (Apaf 1) and, therefore, in caspase activation [38,39,40]. On the other hand, the chemical modification of the Cyt c NP surface did not significantly affect the residual activity of Cyt c, retaining 88–96% of its caspase activity. This effect could be attributed to a surface-protein modification only, while most of the Cyt c remained intact in the core of the NP. Thus, Cyt c activity in the Cyt c-PLGA-PEG-FA NP formulation is better preserved than in NP-free formulation Cyt c-PEG-FA.

It has been reported that DI water nanoprecipitation of Cyt c (10 mg/mL) in the presence of methyl-β-cyclodextrin, using acetonitrile as co-solvent (DI water:acetonitrile ratio 1:4), followed by surface decoration with the co-polymer PLGA-PEG-FA, results in Cyt c-PLGA-PEG-FA NPs with a diameter of 338 ± 8 nm [12]. We followed a similar methodology for both the polymer and NPs synthesis. The peaks in the SH-PLGA-PEG-FA NMR spectrum were in agreement with the previous reports. Several parameters during the nanoprecipitation procedure were tested to decrease NP size and enhance NP penetration into the tumor, but only alterations in the initial Cyt c concentration led to NP diameter reduction. The correlation of protein concentration with NP diameter, PDI, and precipitation efficiency during nanoprecipitation has shown to be a tendency for other proteins as well [16,41]. Another approach to reduce Cyt c-PLGA-PEG-FA NPs diameter in our system could be employing a polymer with lower molecular weight.

Cell viability studies showed that NP formulations, especially NP-253, are more cytotoxic than the NP-free formulation Cyt c-PEG-FA, showing a lower IC_50_ (49.2 versus 129.5 μg/mL, respectively). This could be attributed to the lower caspase activation residual activity of Cyt c-PEG-FA. The larger loading capacity of Cyt c in the NPs may be adding to the higher toxicity of the formulation. In the case of the NPs, a higher amount of Cyt c can enter the cells with each FR internalized. Fluorescence studies confirmed that both types of formulations (NP-free and NP-containing) were internalized by LLC cells, but the NPs were about 3 times more effective at inducing cell death. Studies from our lab have shown that this internalization is mediated explicitly by the FR [12,15]. In these studies, it was confirmed that cells treated with the optimized Cyt c-PLGA-PEG-FA NPs were undergoing an apoptotic cell death [42]. One detail we must also consider is that Cyt c from horse heart was used, which has approximately 83% identity with its human counterpart [43]. The use of human Cyt c or a more effective/stable recombinant mutant may help broaden the therapeutic potential of this drug delivery system.

In our in vivo studies, infrared-labeled NPs (NP-253) followed the classical pattern of intravenously injected drugs, showing increased IR intensity in highly vascularized organs such as lung and heart, brain, and tumor, within the first 5 min after injection, and decreasing the IR intensity in 1 h, proving that this system can work in vivo. Based on the IR intensity of the NP in the organs throughout time, these have already started their metabolism after 1h of circulation in the system, as their signal mostly distributes in kidneys and liver at this time point and continues after 6 h. These NPs were not immediately degraded as they went into the blood, standing undetected and biocompatible in an immune-competent mouse model for at least 6 h. Even though the NP reached places such as the brain, heart, lung, kidneys, and liver, the mice showed no sign of toxicity after injection. Long-term exposure studies of mice and pathological analysis of tissues after treatment with NPs are ongoing. Finally, the NP’s capability of binding to the tumor opens the way to the development of this NP formulation as a real treatment against non-small lung cell carcinoma, following the steps of drugs such as Abraxane^®^, a current NP-based, FDA-approved medication to treat lung cancer [28]. Continuing studies aim towards the development of a low-dose, non-toxic, targeted drug delivery system using the optimized Cyt-c nanoparticle formulation to treat lung carcinoma either alone or as adjuvant treatment in vivo.

## 4. Materials and Methods

### 4.1. Materials

#### 4.1.1. Constructs and Nanoparticle Synthesis and Decoration

Cytochrome c from equine heart ≥ 95%, α-Lactalbumin from bovine milk, acetonitrile, ethanol, acetone, dimethylformamide (DMF), reduced glutathione ethyl ester, N,N-diisopropylethylamine (DIEA), tributhylphosphine (PBu3), and 3-nitro-2-pyridinesulfenyl chloride (Npys-Cl) were purchased from Sigma-Aldrich (St. Louis, MO, USA). Carboxylic PEG acid, mPEG-COOH (2000 Da), Folic acid PEG thiol, Folate-PEG-SH (2000 Da), and Folic acid-PEG-FITC (MW 3400 Da) were purchased from NanoCS (Boston, MA, USA). Folate-poly(ethylene glycol)-carboxylic acid FA-PEG-COOH (MW 3000 Da) and Poly(lactide-co-glycolide)-thiol end cap (Mn: 10,000–30,000 Da) were purchased from Akina, Inc. (West Lafayette, IN, USA). Succinimidyl-3-(2-pyridyldithio) propionate (SPDP) was obtained from Thermo Fisher Scientific (Waltham, MA, USA). Fluorescein isothiocyanate isomer I (FITC) was purchased from Sigma-Aldrich (St. Louis, MO, USA).

#### 4.1.2. In Vitro Immunofluorescence Studies and Cell Uptake of FITC-Labeled NPs

Fluoroshield histology mounting medium was purchased from Sigma-Aldrich (St. Louis, MO, USA). DAPI (NucBlue^®^) was obtained from Thermo Fisher Scientific (Waltham, MA, USA). CF™ Dye Phalloidin Conjugates were purchased from Biotium, Inc. (Fremont, CA, USA). Cell lines LLC cells (ATCC CRL-1642), MRC-5 (ATCC CCL-171), NIH/3T3 (ATCC CRL-1658), and HeLa (ATCC CCL-2) were all purchased from ATCC company.

#### 4.1.3. Caspase Activity and Cell Viability Assays

CasPASE Apoptosis Colorimetric Assay (caspase 3, 7, and 10) was purchased from G-Biosciences (St. Louis, MO, USA). CellTiter 96 aqueous non-radioactive cell proliferation assay was purchased from Promega Corporation (Madison, WI, USA).

#### 4.1.4. In Vivo Studies

C57BL/6J mice were purchased from Jackson Labs. LLC cells (ATCC CRL-1642) and ECM Growth factor reduced gel were from Engelbreth-Holm-Swarm murine sarcoma (SIGMA). Near-infrared reactive dye IRDye^®^ 680RD was available as a protein Labeling Kit-High Molecular Weight from LI-COR Biosciences. D-Trehalose was obtained from Acros Organics.

### 4.2. Constructs and Nanoparticle Synthesis and Decoration

#### 4.2.1. Synthesis of Cyt c-PEG-FA (Nanoparticle-Free Formulation)

Different equivalents of SPDP (following Table 1, 0.93, or 1.95 moles of SPDP) were dissolved in 10 µL of acetonitrile and added to 1 mL of 2 mg/mL Cyt c in PBS-EDTA. The solution was stirred at room temperature for 30 min. The unreacted SPDP was extracted using 10 KDa Amicon Ultra filters. The resulting Cyt c-SDPD was reacted with SH-PEG-FA overnight. The unreacted SH-PEG-FA was separated using 10 KDa Amicon Ultra filters. The product Cyt c-PEG-FA was generated and stored in aliquots at −80 °C.

#### 4.2.2. Protein Nanoprecipitation

Cyt c NPs were obtained using a solvent displacement method. Cyt c was dissolved in DI water at a concentration of 2.5, 5, and 10 mg/mL. Subsequently, the anti-solvent acetonitrile was added at a constant rate of 120 mL/h using an automated syringe pump while constantly stirring at room temperature, keeping a ratio of at least 1:4 DI water:acetonitrile.

#### 4.2.3. Determination of Precipitation Efficiency

After nanoprecipitation, the Cyt c NPs suspension was centrifuged for 10 min at 6.2× *g* at room temperature. The supernatant was discarded, and the pellet was lyophilized. The dry NPs were resuspended in PBS 10 mM pH 7.4, and the concentration was determined by measuring the absorbance at 530 and 410 nm using a multimode microplate reader. Precipitation efficiency (PE) was calculated using the following formula:

$$PE=\frac{mg\;of\;Cyt\;c\;in\;pellet\;}{Initial\;mg\;of\;Cyt\;c}\times 100$$

#### 4.2.4. Synthesis of SH-PLGA-PEG-FA

The thiol group in SH-PLGA (MW) was protected with NPys-Cl in anhydrous DMF at RT using 3.3 mg of NPyc-Cl per each 500 mg of SH-PLGA. The product (Nyps-PLGA) was dialyzed against DI water and lyophilized. PLGA-Nyps (400 mg) were mixed with 120 mg of COOH-PEG-FA in anhydrous DMF in the presence of an excess of DIEA. The reaction was stirred overnight under an argon atmosphere. The product (Nyps-PLGA-PEG-FA) was dialyzed against DI water and lyophilized. The Nyps-PLGA-PEG-FA thiol group was deprotected using an excess of PBu3 in a reaction left overnight at RT in anhydrous DMF and an argon atmosphere. The final product, SH-PLGA-PEG-FA, was dialyzed against DI water and lyophilized.

#### 4.2.5. Synthesis of Cyt c-PLGA-PEG-FA Nanoparticles

After nanoprecipitation, the crosslinker SPDP was added to the Cyt c NP suspension and stirred for 30 min at room temperature. A 1:8 Cyt c-to-linker molar ratio was used. The co-polymer SH-PLGA-PEG-FA was subsequently dissolved in added acetonitrile. The reaction was stirred overnight at room temperature. Cyt c-PLGA-PEG-FA NPs were centrifuged 10 min 6.2× *g* at room temperature and washed three times with DI water.

### 4.3. Physicochemical Characterization of Nanoparticles

#### 4.3.1. ^1^H NMR

^1^H NMR spectra of SH-PLGA-PEG-FA co-polymer were acquired with a Bruker 700 FTNMR spectrometer. Spectra were obtained on DMSO-d^6^ solutions in 5 mm diameter tubes, and chemical shifts were quoted in parts per million relative to the residual signal of DMSO (δH = 2.50 ppm). The spectra were processed using Mestrenova (Mnova 11.0 Mestrelab Research) software.

#### 4.3.2. Dynamic Light Scattering

To determine the diameter and PDI of Cyt c and Cyt c-PLGA-PEG-FA NPs, these were resuspended in acetonitrile and DI water, respectively. For zeta potential determination, Cyt c-PLGA-PEG-FA NPs were resuspended in DI water and transferred to folded capillary cells. Diameter, PDI, and zeta potential were obtained using a zetasizer nano from Malvern (Westborough, MA, USA).

#### 4.3.3. Scanning Electron Microscopy

Lyophilized NPs were coated with gold for 50 s (10 nm) using a Pepco SC-7 auto sputter coater. Images of coated samples were obtained using a scanning electron microscope at 20 kv. The SEM model used was a JEOL 6330F Field Emission Scanning Electron Microscopy (FEG-SEM) at the Florida International University’s EM Facility.

#### 4.3.4. Determination of Encapsulation Efficiency and Actual Loading

After the overnight reaction for Cyt c-PLGA-PEG-FA NPs synthesis, NPs were centrifuged for 10 min, RT at 6.2× *g*. The NPs were washed by resuspending the pellet in DI water and centrifuging three times, 10 min, RT at 6.2× *g*. NPs were lyophilized, and the total yield (mg of dry Cyt c-PLGA-PEG-FA NPs obtained) was determined. The concentration of Cyt c in the supernatants was determined by measuring the absorbance at 530 and 410 nm using a multimode microplate reader. Encapsulation efficiency (EE) and actual loading (AL) were calculated using the following equations:

(1) $$\mathrm{EE}:\;\frac{Initial\;amount\;of\;Cyt\;c-Cyt\;c\;in\;supernatant}{Initial\;amount\;of\;Cyt\;c\;}\times 10$$

(2) $$AL=\frac{mg\;of\;Cyt\;c\;in\;nanoparticles\;}{mg\;of\;nanoparticles}\times 100$$

#### 4.3.5. Study of the Release Profile of Cyt c-PLGA-PEG-FA NPs

Both NP formulations, NP-253 and NP-354, were resuspended at a concentration of 0.5 mg/mL in PBS buffer containing different amounts of GHS (0, 0.001, and 10 mM). Resuspended NPs were incubated at 37 °C for different times: 0.5, 1, 3, 5, 10, 20, and 46 h. After each time point, the suspension was collected and centrifuged for 10 min at 13.8× *g*. The supernatants were separated from the pellet to determine the Cyt c concentration released in the supernatant. The pellet was resuspended in the respective buffer and returned to incubation. The Cyt c concentration in the supernatant was determined through UV–Vis spectroscopy using a Synergy^TM^ multi-plate reader. The presence of a reducing agent like GHS produces a shift in Cyt c’s absorbance peak within the visible region of the spectrum. Therefore, UV–Vis scans were performed to measure Cyt c absorbance for each condition, and a calibration curve was constructed for each of the release conditions tested. The possible presence of folic acid in the supernatant (which absorbs ~410 nm) from the NPs was considered, and the maximum absorbance where no interference of FA could be detected was used. Thus, the wavelength to calculate the release of non-reduced Cyt c when incubated in PBS with 0 and 0.001 mM GHS was 530 nm, while the wavelength to calculate the release of reduced Cyt c incubated in PBS containing 10 mM GHS was at 550 nm. This experiment was performed in triplicate, and the results were used to calculate a cumulative release profile curve.

### 4.4. In Vitro Cell Studies to Characterize Constructs and NPs

#### 4.4.1. Study of the Kinetics of Folic Acid Uptake by LLC Cells

Lewis Lung Carcinoma cells were grown in 24-well plates at a density of 50,000 cells/well and incubated overnight. Cells were added to 50 µM Folate-PEG-FITC or mPEG-FITC in RPMI medium. After different incubation times, cells were washed with PBS, fixed with 4% paraformaldehyde, and left for 20 min at RT. Subsequently, cells were incubated with DAPI (NucBlue^®^) nuclear stain (Ex. 358 nm, Em. 361 nm) for 5 min at RT. In Figure 2, an additional actin stain was added to label the cytoskeleton (CF™ Dye Phalloidin Conjugates, Ex. 562 nm, Em. 583 nm), and left for 20 min at RT. After washing three times for 5 min with PBS, fixed and stained cells were mounted in a microscopy slide using fluoroshield mounting media and analyzed in a confocal microscope (NIKON eclipse) using a 60× objective. The intensity of the images obtained was measured using the program Image J.

#### 4.4.2. Cell-Free Caspase 3, 7, and 10 Assay

Cell lysate, samples, and assay were prepared following the protocol of the CasPASE Apoptosis Colorimetric Assay (caspase 3, 7, and 10) from G-Biosciences (St. Louis, MO, USA). Briefly, 5 × 10^6^ HeLa cells were resuspended in 100 µL of lysis buffer, flash frozen for 2 min, and thawed in a 37 °C bath. After four cycles of freezing/thawing of cells, these were centrifuged for 20 min at 4 °C, 11,000 rpm. The supernatant (lysate) was used to continue with the assay. Cell lysate was activated by mixing with a 0.2 mg/mL Cyt c solution in a volume ratio 1:1 and incubated at 37 °C for 150 min. In a 96-well plate, 10 µL of active lysate was mixed with 10 µL of substrate labeled with the chromophore ρ-nitroanilin (Ac-DEVD-ρNA) (2 mM) and 80 µL of caspase assay buffer. The plate was incubated at 37 °C, and the absorbance at 405 nm was monitored periodically in an absorbance plate reader.

#### 4.4.3. In Vitro Cell Viability Assay

LLC cells were grown in a 96-well plate at a density of 10,000 cells/well. After overnight growth, cells were treated with different Cyt c formulations at different concentrations, as well as their respective controls. The MTS (3-(4,5-dimethylthiazol-2-yl)-5-(3-carboxymethoxyphenyl)-2-(4-sulfophenyl)-2H-tetrazolium) cell viability assay from Promega was used and measured in an absorbance plate reader at 490 nm to determine the cell viability after treatment, following instructions from the kit manufacturer.

#### 4.4.4. Calculation of IC_50_ for NP-Free Cyt c-PEG-FA Formulation and Surface-Decorated NP-253 and NP-354 Formulations

To calculate the IC_50_ for the NP-free Cyt c-PEG-FA and the NP formulations, NP-253 and NP-354, LLC cells were used, and an MTS assay was performed following the procedure described above in Section 4.4.3 at the following concentration range for Cyt c-PEG-FA: 15, 30, 60 and 120 μg/mL; and for the NP-253 and NP-354 formulations: 22.5, 45, 90 and 180 μg/mL. The percentiles of viability were transformed to a logarithmic scale using the functions X = Log(X) in the program Graph Pad Prism, and the Y values were normalized (being 0% the smallest value in the data set and 100% the highest value in the data set). Finally, a non-linear fit of drug inhibition (log (inhibitor) vs. normalized response- variable slope) was performed to obtain the best fit IC_50_ and the R-squared values. Three experimental replicate results were used for each formulation.

#### 4.4.5. Study of Apoptosis Induction Using Propidium Iodide

LLC cells were incubated with 35 µg/mL of Cyt c-PLGA-PEG-FA, and after 6 h these were washed with PBS and incubated with propidium iodide (PI) for 5 min at 37 °C. Cells were fixed for 20 min at RT using 4% paraformaldehyde and incubated with DAPI (NucBlue^®^) for 5 min. After three washes, cells were mounted using fluoroshield mounting media. Control (untreated) cells were exposed to the same procedure, without the Cyt c-PLGA-PEG-FA incubation.

#### 4.4.6. Study of Formulations for NP Internalization

LLC cells were incubated with 35 µg/mL of Cyt c-PLGA-PEG-FA NPs (NP-253) and after 6 h washed with PBS, fixed for 20 min at RT using 4% paraformaldehyde, and incubated with DAPI (NucBlue^®^) for 5 min. After three washes with PBS, cells were laid under a coverslip using fluoroshield mounting media. Untreated cells were used as control. F-Actin cytoskeleton in cells was labeled using CF™ Dye Phalloidin Conjugates.

### 4.5. In Vivo Studies to Detect Near-Infrared NPs in Mice

#### 4.5.1. Cell Growth and Induction of Lewis Lung Carcinoma in Mice

*Cell culture of Lewis Lung carcinoma cells for tumor implant*: LLC cells (ATCC CRL-1642) were cultured in High-Glucose Dulbecco’s Modified Eagle’s Medium (DMEM) supplemented with 10% fetal bovine serum (FBS) and 1% penicillin/streptomycin/amphotericin (PSA) to confluency, and these were gently scraped off the plate, pelleted, and quantified. Mice were subcutaneously injected with 1 × 10^7^ LLC cells to induce tumor growth, in a 400 μL total volume composed of 200 μL ECM Growth factor reduced gel from Engelbreth-Holm-Swarm murine sarcoma (SIGMA), and 200 μL cell suspension in media into the upper right dorsal area of their body.

*Mice*: Eight-week-old C57BL6J male mice weighing 25–28 g were used. These mice were injected with labeled NPs at an average of 10 d after tumor implant. Before injection, mice were anesthetized with 1% isoflurane, and a tail vein injection of 0.5 mg of labeled NPs with IRDye 680RD was administered in a volume of 200 μL. Mice were euthanized after 5 min, 1 h, or 6 h. Tumor and organs were extracted and scanned for NP distribution using the LI-COR Odyssey CLx infrared scanner. The content is solely the responsibility of the authors and does not necessarily represent the official views of the National Institutes of Health. All necessary approvals from the Institutional Animal Care and Use Committee (IACUC) were in place for the performed research: Assurance ID number: D16-00343, IACUC Protocol Universal Number: 048-2018-15-01.

#### 4.5.2. Labeling of Nanoparticles with a Near-Infrared Dye

To detect NPs in tissue, these were labeled with the near-infrared reactive dye IRDye^®^ 680RD (available as a protein Labeling Kit-High Molecular Weight from LI-COR Biosciences). In this reaction, an NHS ester (amine-directed) nanoparticle-IRDye crosslink reaction was performed following manufacturer instructions. The steps involving the 680RD dye were performed protecting materials from light. In general, the procedure carried with our NPs was the following: Cyt c-PLGA-PEG-FA nanoparticles used in the in vivo experiments had a diameter of 253 ± 55 nm as determined by DLS. These NPs had a composition of 62% of Cyt c and were resuspended in PBS to reach a final Cyt c concentration of 1 mg/mL. NPs were sonicated for 20 min to resuspend. IRDye680 (0.1 mg) was resuspended in DI water to a final concentration of 4 mg/mL. Dye and DI water were vortexed to ensure full resuspension. Dye prepared was added to Cyt c-PLGA-PEG-FA NPs, and these were incubated for 2 h at RT. After the reaction time, NPs were centrifuged 15 min at 6.2× *g* at RT. The supernatant was collected, and the pellet was resuspended in PBS to wash. A second centrifugation was made for 15 min at 8000 rpm. The washing step was repeated twice. After the second wash with PBS, the pellets containing the labeled nanoparticles were frozen and lyophilized.

#### 4.5.3. Nanoparticle Optimization for In Vivo Tumor Targeting

To optimize the in vivo targeting of the tumors, the excipient trehalose was added to NPs to improve their solubility in PBS. To perform co-lyophilization of NPs and trehalose, they were first synthesized by nanoprecipitation. After an overnight synthesis reaction, Cyt c-PLGA-PEG-FA NPs were centrifuged for 10 min, speed of 6.2× *g*, at RT. The supernatant was discarded, and the pellet resuspended in 1 mL of a trehalose solution (2 mg/mL). The centrifugation and resuspension of the pellet were repeated twice to wash the NPs. After a third centrifugation, NPs were resuspended in 1 mL of a trehalose solution containing 1:2 nanoparticles:trehalose weight ratio and lyophilized as a suspension in this solution.

*NPs diameter determination*: After NPs were lyophilized, these were resuspended in DI water and sonicated for 5 min. The diameter was determined using Dynamic Light Scattering (DLS). The results are the average of three runs, and the Cyt c-PLGA-PEG-FA NPs used in this in vivo assay had a diameter of 253 nm.

#### 4.5.4. Detection of Infrared-Labeled Nanoparticles In Vivo

NPs detection in tissues was made using the LI-COR Odyssey CLx near-infrared imaging system. A 169 μm resolution and a medium quality setting were chosen in the Image Studio^TM^ imaging software. All the tissue area was selected for quantification, and the IR signal was obtained using the Image Studio^TM^ software. The following formula was used to obtain the percentage of IR signal of the IR-NP-253-exposed tissue over the control tissue:

% IR signal of NP-injected tissue over control tissue: [$\frac{IR\;signal\;from\;NP\;injected\;mouse}{IR\;signal\;of\;control\;mouse}\times 100$] − 100.

## 5. Conclusions

We confirmed the superiority of nano-sized NP formulations over individual Cyt c tagged with FA. Our optimized NP formulation retained 88–96% of Cyt c activity, inducing apoptosis and decreasing lung carcinoma cell viability with a lower IC_50_ than the NP-free formulation. A reduction of around 100 nm (30%) in NP diameter was achieved, enhancing the previously reported system, which had a diameter of 338 ± 8 nm. The optimized Cyt c PLGA-PEG-FA NPs showed selective cytotoxicity towards non-small cell lung carcinoma cells overexpressing FR, including LLC and HeLa, but not towards normal cells. In vivo studies showed that these NPs are biocompatible, immune-system resistant, they can quickly bind to LLC tumors 5 min after application by the intravenous route, and remain in circulation at least 6 h. These results open way to the in vivo testing of this NP formulation as a targeted drug delivery system for the treatment of lung carcinoma or other FA-overexpressing tumors.

## Figures and Tables

**Table 1:** Cytochrome c modification with SPDP at different molar ratios of Cyt-c-to-linker ^a^.

Molar Ratio Cyt c to SPDP	Moles of SPDP Per Mole of Cyt c
1 to 2	0
1 to 4	0.93 ± 0.06
1 to 8	1.95 ± 0.34

^a^ Reaction conditions: 2.0 mg/mL Cyt c in PBS-EDTA, pH 7.4, incubated with increasing amounts of SPDP at room temperature for 30 min. Reactions prepared for each condition *n* = 3.

**Table 2:** Different anti-solvents used to nanoprecipitate Cyt c ^a^.

Solvent	Particle Diameter (nm)
Acetonitrile	163 ± 13
THF	219 ± 9
Acetone	No Cyt c precipitation
Ethanol	No Cyt c precipitation

^a^ Reaction conditions: 5 mg/mL Cyt c in DI water was used. Solvent:anti-solvent ratio of 1:4. The anti-solvents were added a constant rate of 120 mL/h under constant stirring.

**Table 3:** Effect of Cyt c concentration during nanoprecipitation on NP diameter, PDI, precipitation efficiency, and relative caspase 3, 7, and 10 activation ^a^.

Cyt c Concentration (mg/mL)	Diameter (nm) ^b^	PDI ^b^	Precipitation Efficiency (%) ^b^	Relative Caspase Activation (%) ^b^
2.5	139 ± 15	0.041 ± 0.03	33.0 ± 7.2	104 ± 10
5	163 ± 13	0.064 ± 0.03	71.9 ± 8.0	96 ± 3
10	251 ± 7	0.13 ± 0.08	84.5 ± 12.3	88 ± 2

^a^ Reaction conditions: Nanoprecipitation of Cyt c at different protein concentrations using DI water as solvent and acetonitrile as anti-solvent. Solvent:anti-solvent ratios used were 1:8, 1:4, and 1:4 for 2.5, 5, and 10 mg/mL Cyt c, respectively. These NPs did not have any surface modification. ^b^ Polydispersity index, PDI, is a measure of particle size dispersion (the lower the PDI, the more homogeneous the size of the NPs). Data are the averages and SD of three different batches of NPs.

**Table 4:** Parameters of Cyt c-PLGA-PEG-FA NPs obtained using 5 mg/mL (NP-253) and 10 mg/mL (NP-354) Cyt c during nanoprecipitation. The same stoichiometries (molar ratios of Cyt c, crosslinker, and SH-PLGA-PEG-FA) were used in both cases ^b^.

	Nanoparticle Name	Cyt c NP ^a^	NP-253	NP-354
Parameter				
Cyt c used during nanoprecipitation (mg/mL)		2.5	5	10
Diameter before coating with PLGA-PEG-FA (nm)		139 ± 15	163 ± 13	251 ± 7
Diameter after coating with PLGA-PEG-FA (nm)		-	253 ± 55	354 ± 11
PDI		-	0.07 ± 0.06	0.13 ± 0.03
Encapsulation efficiency (%)		0	45 ± 7	48 ± 9
Actual loading (%)		-	62 ± 10	66 ± 10
Relative caspase activity (%)		-	89 ± 9	99 ± 6
Zeta potential (mV)		-	26.9 ± 5.03	22.4 ± 6.36

^a^ The use of 2.5 mg/mL Cyt c during the nanoprecipitation did not yield Cyt c-PLGA-PEG-FA NPs. ^b^ NP data in the table are the averages of three batches prepared and the respective SD. Parameters of diameter, PDI, and zeta potential were obtained by DLS. Cyt c concentration, encapsulation efficiency, actual loading, and relative caspase activity were obtained by UV–Vis spectroscopy.
